# Occupational diseases among dental personnel: a scoping review

**DOI:** 10.25122/jml-2024-0412

**Published:** 2025-06

**Authors:** Florentina Căminișteanu, Andrei Vorovenci, Viorel Ștefan Perieanu, Sergiu-Adrian Petri, Liliana Burlibașa, Mihai David, Andi Ciprian Drăguș, Mihai Burlibașa

**Affiliations:** 1 Department of Dental Technology, Faculty of Midwifery and Nursing, Carol Davila University of Medicine and Pharmacy, Bucharest, Romania; 2 Doctoral School, Carol Davila University of Medicine and Pharmacy, Bucharest, Romania; 3 Prosthodontics Residency, Carol Davila University of Medicine and Pharmacy, Bucharest, Romania; 4 Faculty of Medicine, Lucian Blaga University of Sibiu, Sibiu, Romania; 5 Department of Genetics, University of Bucharest, Faculty of Biology, Bucharest, Romania

**Keywords:** occupational diseases, dentistry, musculoskeletal disorders, burnout, allergy, hearing loss, respiratory diseases

## Abstract

Dental professionals face numerous occupational health risks that can significantly impact their well-being and career longevity. This scoping review synthesizes current evidence on the prevalence, risk factors, and prevention strategies for major occupational health issues in dentistry. The article selection process adhered to the Preferred Reporting Items for Systematic Reviews and Meta-Analyses (PRISMA) guidelines. A comprehensive electronic search was conducted across PubMed/MEDLINE, Scopus, and Web of Science to identify relevant studies published within the past decade. Musculoskeletal disorders (MSDs) were found to be highly prevalent. Allergic contact dermatitis (ACD), pulmonary diseases such as pneumoconiosis, and noise-induced hearing loss (NIHL) remain common concerns for the overall health of dental personnel. Work-related stress is widespread and can lead to mental health issues such as burnout syndrome, emotional exhaustion, and suicidal ideation. Occupational health issues are prevalent in dentistry, necessitating the development of improved prevention strategies. Recommended preventive measures include ergonomic workplace design, regular physical activity, stress management techniques, the use of appropriate personal protective equipment, and efficient ventilation systems. Future research should focus on developing standardized diagnostic criteria and employing prospective cohort designs to more accurately estimate disease burden and evaluate the effectiveness of interventions.

## INTRODUCTION

Due to the advent of new technologies and protocols in recent years, the dental practice, as a whole, is changing at a rapid rate. Dental personnel (dentists, assistants, dental technicians) are compelled to adapt to different working conditions, arguably healthier than those experienced by their predecessors. Nonetheless, a wide range of occupational risks and hazards persist in the working environment, affecting the livelihood of numerous professionals in this field. From musculoskeletal disorders (MSDs) to stress and burnout, the literature is rich in various maladies affecting dental personnel, and the aim of this overview was to provide a general understanding of the main work-related issues relevant in the dental world today [1-6]. Specifically, this review addresses the following themes: MSDs, allergic contact dermatitis (ACD), respiratory diseases among dental technicians, noise-induced hearing loss (NIHL), and stress-related disorders. Ergonomics, defined as the applied science of designing and arranging workplaces to optimize human well-being and overall system performance, is central to understanding and mitigating these risks [7]. Among the various occupational hazards, MSDs remain the most prevalent across different regions globally [8-10]. Several systematic reviews, studies, and case reports have been published, outlining the extensive impact and the measures that should be taken to reduce it [3,9,11,12]. Methacrylate and latex sensitivities are a growing concern in the workplace. Although the introduction of alternative protective gear can be a solution, recent studies show that sometimes, more than half of dental professionals (doctors, students, technicians) develop lesions related to latex and methacrylate [13-15].

Respiratory illnesses, particularly pneumoconiosis, represent a significant occupational hazard for dental technicians [16,17]. This fibrotic lung disease results from prolonged inhalation of airborne particulate matter, especially silica dust generated during sandblasting procedures [18]. Clinical manifestations typically include chronic dry cough and exertional dyspnea, and in severe cases, complications may progress to tuberculosis or even lung cancer [19].

Noise-induced hearing loss is another critical yet often underestimated occupational hazard [20-26]. Exposure to long or repeated sounds exceeding 85 dB has adverse effects on auditory health. A systematic review published in 2023 further emphasized the importance of recognizing and mitigating this risk in dental settings [22].

Ultimately, dentistry is widely recognized as a high-stress profession, with numerous contributing factors, including patient management, clinical workload, regulatory pressures, and personal stressors. Chronic occupational stress can culminate in burnout, a syndrome characterized by physical and emotional exhaustion, reduced energy levels, and professional dissatisfaction. The prevalence of stress and burnout varies by role, region, and workplace culture [27,28].

## MATERIAL AND METHODS

A comprehensive literature search was conducted across three major electronic databases: PubMed/MEDLINE, Scopus, and Web of Science. The search included studies published between January 1, 2014, and October 15, 2024, and was conducted in accordance with the Preferred Reporting Items for Systematic Reviews and Meta-Analyses (PRISMA) guidelines. The keywords and MeSH Terms used in the online research were: 'occupational diseases' [MeSH Terms] AND 'dental staff' [MeSH Terms] OR 'dental personnel', 'allergy' OR 'hypersensitivity' [MeSH Terms] AND dentists' [MeSH Terms] OR ('dental technicians' [MeSH Terms], 'occupational exposure' [MeSH Terms] AND 'dental staff' [MeSH Terms]). Selection criteria included articles published within the last 10 years that presented data on occupational diseases among dental personnel. Eligible study types encompassed longitudinal studies, case-control studies, cohort studies, and both narrative and systematic reviews. Case reports, animal studies, and in vitro studies were excluded. Searches were conducted both electronically and manually, and EndNote X9 (Clarivate, 2013; Philadelphia, PA) was used to manage references and remove duplicates.

## RESULTS

**Figure 1:**
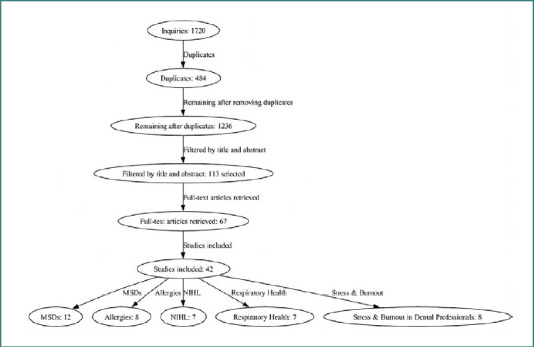
PRISMA flowchart of the screening process

The initial database search yielded 1,720 articles. After removing 484 duplicates, 1,236 articles remained for title and abstract screening. From these, 113 studies were selected for full-text review, and 67 full-text articles were successfully retrieved. Finally, 42 studies met the inclusion criteria and were included in the final analysis. These consisted of 12 studies on MSDs, eight on allergic conditions, seven on NIHL, seven on respiratory diseases, and eight on stress and burnout among dental professionals (Figure 1). A scoping review approach was selected due to the considerable heterogeneity observed in the literature, with a wide range of risk factors and diseases being examined within the field of dentistry, hence allowing for a broader exploration of the existing body of knowledge and facilitating a comprehensive understanding of the various topics without narrowing the focus [29,30]. The diverse nature of the studies, encompassing different populations, methodologies, and outcome measures, made conducting a systematic review a challenging task. Given the diversity of included studies and the aim of summarizing rather than critically appraising individual study quality, a formal risk of bias assessment was not performed [29,31].

### Musculoskeletal disorders

**Figure 2:**
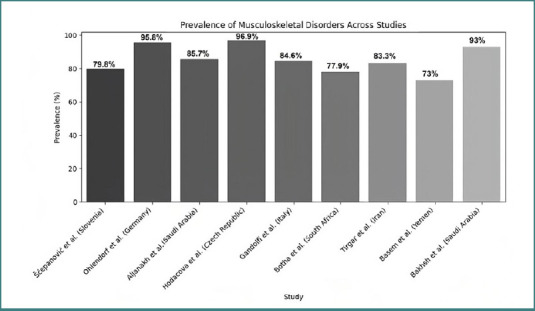
Prevalence of musculoskeletal disorders across studies

**Figure 3:**
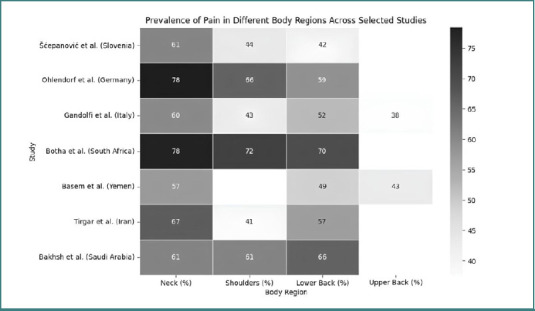
Prevalence of pain in different body regions in the included studies

Musculoskeletal disorders are among the most prevalent occupational health issues affecting dental professionals globally. In Germany, a study found that 92% of dentists and dental students experienced MSDs in the last 12 months, with a lifetime prevalence of 95.8%. The most affected regions were the neck (78.4%), shoulders (66.2%), and lower back (58.7%), while pain was generally reported on the right side of the body [32]. Czech dentists reported similar patterns, with 96.9% of respondents experiencing at least one kind of musculoskeletal problem in the past 12 months. The prevalence rates for moderate or major-intensity pain were highest for the lower back, followed by neck and upper back pain, shoulder complaints, and headaches [33]. In Slovenia, 79.8% of dental workers reported at least one MS complaint, the most common symptoms including pain, which was most frequently experienced in the neck (60.7%), lower back (41.7%), and right shoulder (44.0%) [34]. In Italy, 84.6% of dental professionals were affected by MSDs in the last 12 months, the most affected areas being the neck (59.9%), lumbar region (52.1%), shoulders (43.3%), dorsal region (37.7%), and wrists (30.6%) [5]. In a study from Saudi Arabia, 93% of dental professionals reported MSDs in at least one body site within the past year, with the lower back (66%), shoulders (61%), and neck (61%) being the most commonly affected areas. A study focusing only on dental assistants from the same region reported a high prevalence of MSDs, with 85.7% experiencing symptoms during the past 12 months and 47.9% during the past seven days, the most affected body regions being the shoulders, followed by the lower back, upper back, and neck [35]. An Iranian study by Tirgar *et al*. reported that 83.3% of dentists experienced musculoskeletal pain, with the neck (67%), lower back (56.7%), and shoulders (41%) being the most affected areas. Similarly, in Yemen, musculoskeletal disorder (MSD) prevalence was high, with pain most frequently reported in the neck (57.3%), lower back (48.9%), upper back (43.1%), shoulders, as well as the hands and wrists [36]. In South Africa, a 12-month prevalence of musculoskeletal complaints was noted in the neck (77.9%), shoulders (72.4%), and lower back (69.8%) [37]. While the overall prevalence of MSDs was high across all regions studied, variations were observed in the specific body areas affected and the reported prevalence rates (Figure 2). The neck and lower back consistently appeared as the most affected areas across all regions. However, shoulder pain appeared more prevalent in European countries compared to the Middle East (Figure 3). A study from Slovenia reported a significant prevalence of pain in the hips and buttocks (29.8%), which was not prominently mentioned in studies from other regions. These variations might be due to differences in work practices, ergonomic awareness, or reporting methods across different countries and regions. Several risk factors contribute to the high prevalence of musculoskeletal disorders among dental professionals [34]. Work-related factors play a crucial role, with prolonged static postures, repetitive movements, and precision tasks being significant contributors [5,34]. Dental work often involves neck inclination/rotation, forward bending, and raised arms working in prolonged static isometric/eccentric contraction [5,9]. Working hours also impact MSD prevalence, with a higher risk for operators working more than five hours per day and 30 hours per week [32]. Gender is another important factor, as female dental professionals generally report higher rates of MSDs compared to males [5,32]. The data collected from the included studies are presented in Table 1. Years of experience also influenced MSD prevalence, with some studies finding a higher prevalence among those with 2–5 years of experience [5], while others reported increased prevalence with more years of practice [33]. Furthermore, one study found that a decrease in height among respondents was associated with an increase in neck trouble [37]. The relationship between age and MSD prevalence was less clear, with some studies reporting an increase in MSDs with age, while others found no significant correlation [5]. Psychological factors also play a role, as the perception of work as psychologically demanding was significantly related to neck, lower back, and shoulder pain [33]. In addition, factors such as body mass index (BMI), physical demands during working hours, and awareness of the work environment were found to be significantly associated with MSD occurrence.

**Table 1:** Prevalence, risk factors, and prevention strategies for musculoskeletal disorders across different countries

Study	Country	Prevalence	Risk factors	Prevention strategies
Šćepanović *et al*.	Slovenia	79.8% overall	Prolonged static postures	Ergonomic working environment, regular breaks
Ohlendorf *et al*.	Germany	95.8% lifetime, 92% 12-month, 65.6% 7-day	Female gender	Not specified
Basem *et al*.	Yemen	73% overall	Low ergonomic awareness	Additional training in dental ergonomics
Aljanakh *et al*.	Saudi Arabia	85.7% 12-month, 47.9% 7-day	Age, BMI, physical demands, work environment	Not specified
Hodacova *et al*.	Czech Republic	96.9% overall	Age, gender, length of practice, psychological factors	Not specified
Gandolfi *et al*.	Italy	84.6% 12-month	Working >5h/day, >30h/week	Yoga or stretching, ergonomic education
Botha *et al*.	South Africa	77.9% 12-month	Decrease in height associated with neck trouble	Not specified
Tirgar *et al*.	Iran	83.3% overall	Female gender, age	Regular exercise, ergonomic policy
Bakhsh *et al*.	Saudi Arabia	93% 12-month	Keeping uncomfortable posture for long periods, lifting heavy objects	Increasing the number of employees, enabling regular breaks, reducing the duration of clinical work

### Carpal tunnel syndrome

Among the MSDs, carpal tunnel syndrome (CTS) is a separate, well-documented, and prominent occupational health concern among dental professionals. In a study conducted in Riyadh, Saudi Arabia, 30.5% of dentists reported experiencing mild or severe CTS symptoms, while in another study conducted in Iran, 17.9% of dentists were diagnosed with CTS in at least one hand [38]. This prevalence is higher than that found in the general population, which ranges from 3% to 6% [1,38,39]. A German study revealed that 30.8% of dentists reported MSDs in the hand at any time in their lives, with 20.3% experiencing symptoms in the last 12 months and 9.5% in the last 7 days, while dental assistants showed even higher prevalence rates, with 42.6% reporting hand MSDs in their lifetime, 31.8% in the last 12 months, and 15.3% in the last 7 days [2]. Several risk factors for CTS in dental professionals have been identified. Female gender appears to be a significant risk factor, with female dentists more likely to report symptoms than their male counterparts [2, 39]. This gender disparity may be partly attributed to women's increased pain perception and readiness to report symptoms [2]. One study found that dentists with a BMI of 30 or greater were more likely to complain of CTS symptoms than those of normal weight [39]. However, contradictory findings were reported in an Iranian study, where dentists with carpal tunnel syndrome had a significantly lower mean body mass index (BMI) than those without CTS. Exposure to hand-arm vibration is a significant occupational risk factor for CTS in dentists. Dentists exposed to vibration for more than 2 hours per day had 2.5 times higher odds of developing CTS [38]. Left-handedness was found to be a risk factor in one study, with left-handed dentists significantly more likely to suffer from CTS symptoms [39]. The duration of patient contact also plays a role, with dentists spending more than 8 hours per day with patients being more likely to report CTS symptoms.

### Allergens in the dental profession

Dental professionals face a multitude of occupational hazards, with ACD being a primary concern. Clinically, the symptoms of ACD typically include dry skin, redness, and pruritus [40,41]. Among the causes are several substances, including latex and nitrile, acrylates, disinfectants, and various metals, which have been used over the years. Historically, mercury toxicity in dentistry has been a concern due to the use of amalgam fillings as a source of ACD [42-44]. However, the use of composite resins has become increasingly popular among those seeking tooth-colored fillings, offering an aesthetic alternative that avoids the use of mercury [45]. Latex sensitivity has been a significant issue, with past reports suggesting that 5–25% of dental personnel may be sensitized [46]. This difference between past and current data may be attributed to improved awareness and preventive measures implemented over the years [40,46]. A study conducted in Zagreb, Croatia, found that only 7.0% of dental professionals and students showed positive skin prick test results to latex, while 56.1% of dental professionals reported skin lesions when using latex products [13]. The distinction between irritant and allergic reactions is important for the proper diagnosis and management of occupational skin disorders in dental professionals. The introduction of nitrile gloves as an alternative to latex has not fully resolved skin-related issues, as presented in a study of Bulgarian dentists that revealed that 32.9% of those using only nitrile gloves reported skin disorders, compared to 28.3% of latex glove users [47]. Factors contributing to glove-related skin symptoms include wearing protective gloves for more than 4 hours per day and using more than 10 pairs of gloves daily [13,46,47]. Otherwise, methacrylates and acrylates represent another significant source of allergies in dental professionals (Table 2). A study of dental technicians with occupational contact dermatitis found that 29.6% (67 out of 226) reacted to methacrylates and/or acrylates while the sensitization rates to specific methacrylates varied widely: HEMA (2-hydroxyethyl methacrylate) at 81.1%, HPMA (2-hydroxypropyl methacrylate) at 78.4%, 2-hydroxyethyl acrylate at 54.1%, TREGDA (triethyleneglycol diacrylate) at 43.3%, ethyl acrylate at 37.8%, EGDMA (ethylene glycol dimethacrylate) at 32.4%, and tetraethyleneglycol dimethacrylate at 32.4% [48]. In addition to skin symptoms, recent research has also highlighted potential cellular-level changes in dental professionals exposed to methyl methacrylate (MMA). Thus, a study by Soykut *et al*. found a higher level of buccal-cell anomalies in the exposed group, with statistically significant increases in pycnotic cells, nuclear buds, and micronucleus frequency [49]. Furthermore, metal allergies, while less prevalent than methacrylate allergies, remain a concern in the dental profession. Palladium chloride was identified as the most common metal allergen among dental technicians with occupational contact dermatitis, with a small percentage showing positive reactions. At the same time, all patients who reacted to palladium chloride also reacted to nickel sulfate, suggesting a possible cross-reactivity or co-sensitization [41,48]. Disinfectants pose another significant risk for skin irritation and allergic reactions among dental professionals. In one study, 56.1% of dental professionals reported experiencing work-related skin changes. However, only 6.7% specifically identified soaps and disinfectants as exacerbating factors, suggesting potential underreporting or multifactorial causation in dermatologic symptoms.

**Table 2:** Prevalence of methacrylate allergies in included studies

Author	Allergen/Condition	Prevalence/Incidence
Romita *et al*.	Acrylate sensitivity 2-HEMA detection rate	3.2% (7/217 patients) 100% of acrylate-sensitized patients
Japundžić *et al*.	Self-reported skin lesions Latex allergy (positive SPT)	56.1% (249/444 participants) 7.0% (14/200 tested)
Ramos *et al*.	Occupational ACD cases HEMA detection rate	67.6% of total ACD cases 80.6% of methacrylate cases
Stoeva *et al*.	Work-related skin symptoms Latex glove users with skin symptoms Nitrile glove users with skin symptoms Skin symptoms attributed to soaps/disinfectants	31.6% 28.3% 32.9% 6.7% of those reporting skin changes
Heratizadeh *et al*.	ACD in dental technicians with occupational contact dermatitis ACD in dental technicians without occupational contact dermatitis Methacrylate/acrylate reactivity HEMA sensitivity HPMA sensitivity Ethylene glycol dimethacrylate sensitivity	37.6% 18.5% 29.6% 81.1% of methacrylate-sensitive patients 78.4% of methacrylate-sensitive patients 32.4% of methacrylate-sensitive patients

ACD, Allergic Contact Dermatitis; SPT, Skin Prick Test; HEMA, 2-hydroxyethyl methacrylate; HPMA, 2-hydroxypropyl methacrylate

### Respiratory health

While several studies have examined respiratory conditions across various groups of dental personnel, pneumoconiosis among dental technicians remains the most extensively documented occupational pulmonary disease in dentistry. However, its prevalence varies across studies, being dependent on the type of radiological investigation used [16]. The reported prevalence of pneumoconiosis varies considerably across studies, largely depending on the radiological techniques employed. More recent investigations favor high-resolution computed tomography (HRCT), either alone or in combination with chest X-rays, due to its superior sensitivity in detecting early-stage disease. HRCT has demonstrated greater diagnostic accuracy than chest radiography; in one study, 27% of cases initially classified as normal by chest X-ray were reclassified as Category 1 pneumoconiosis on HRCT [50]. The most common HRCT finding was the presence of round opacities, observed in 38–89.8% of cases [16,51]. Large opacities or progressive massive fibrosis (PMF) were detected in 13.3–21.3% of cases. Other HRCT findings included irregular opacities, ground-glass opacities, emphysema, and pleural abnormalities [16,17,19]. Pulmonary function tests often showed impairment in dental technicians with pneumoconiosis [18,52]. Duration of exposure was associated with an increased risk of pneumoconiosis in some studies [16]. Smoking was not consistently associated with increased risk or severity of pneumoconiosis, although emphysema on HRCT was more common in smokers. Sandblasting was identified as a significant risk factor, increasing the risk of pneumoconiosis 77 times [16]. The high prevalence of pneumoconiosis, even among early-career dental technicians, underscores the need for enhanced workplace protection and regular health screenings [16-19]. Respiratory symptoms were common, with 14.9% of dental technicians reporting respiratory complaints, increasing to 40.8% in those diagnosed with pneumoconiosis [16,50]. In one study, 20.7% of dentists reported current work-related respiratory complaints. Among clinical support staff, latex-attributed rhinitis/conjunctivitis was reported in 25% and latex-induced asthma in 14% of individuals, highlighting that respiratory risks are not exclusive to dental laboratory environments but also extend into clinical practice [47,53].

### Noise-induced hearing loss

The prevalence and risk factors of hearing loss among dental professionals have been a subject of ongoing debate [23,24,54-56]. While some studies suggest a higher risk of NIHL in dentists [21, 25, 26], others indicate that the risk may not differ significantly from that of the general population or other academic professionals [22,54]. A study by Willershausen *et al*. [25] found that dentists had slightly poorer hearing thresholds compared to other academic professionals, particularly at frequencies of 3 kHz and 4 kHz, though these differences were only marginally statistically significant. When bone conduction was measured to assess inner ear integrity, no significant differences were observed between dentists and controls. Several factors may contribute to the risk of hearing loss in dental professionals, including years of experience, equipment usage, and work environment. Some research suggests that dentists with more than 10 years of experience and more than 8 hours of daily work have the highest risk of hearing impairment [22]. The use of high-speed handpieces, ultrasonic scalers, and suction devices has been identified as a primary source of occupational noise for dentists [22,24,57]. The layout and acoustics of dental clinics can also affect noise exposure [54]. A study by Burk *et al*. [20] found that noise levels varied significantly between different clinical settings in dental schools, with the pediatric clinic having the highest average and maximum exposures. The impact of occupational noise exposure may vary across different dental specialties. While general practitioners are exposed to a wide range of dental equipment, the evidence for their increased risk of hearing loss is mixed [25]. Prosthodontists have been reported in one study to have the poorest hearing thresholds at mean frequencies of 500-2000 Hz and 3000-6000 Hz compared to general dentists and dental nurses [22,25,58]. Noise levels may also vary significantly across dental specialties. A study by Burk *et al*. [20] reported that pediatric dental clinics had the highest average and maximum noise exposures among various dental school settings. In some instances, noise levels reached 112.9 dBA during pediatric procedures involving crying children. This suggests that behavioral factors, in addition to equipment, may influence occupational noise risk in certain environments [20]. Dental assistants may be at a heightened risk of noise-related hearing impairment. A study by Al-Omoush *et al*. [21] found that left hearing thresholds were significantly poorer in dental assistants at 1000, 2000, 4000, and 8000 Hz compared to their right ear and reported a significant relationship between the degree of hearing impairment among dental assistants and the daily duration of exposure to dental, occupational noise. While the evidence for significantly elevated rates of NIHL among dentists is mixed, the dental profession does involve exposure to hazardous noise levels. Even if hearing loss rates are not much higher than the general population, dentists report increased annoyance and other auditory symptoms related to occupational noise exposure [23,25,26]. An overview of the data extracted from the included studies is integrated in Table 3. The impact of occupational noise appears to vary across dental specialties, with pediatric dentistry and prosthodontics potentially carrying higher risks [22].

**Table 3:** Key findings from studies on hearing loss

Studies	Main findings
Al-Omoush *et al*.	Statistically significant differences in hearing thresholds between the control group and dental professionals. Left hearing thresholds significantly poorer in dental assistants at 1000, 2000, 4000, and 8000 Hz. Significant relationship between hearing impairment in dental assistants and daily duration of noise exposure.
Myers *et al*.	Dangerous noise levels when high-volume suction used alone or with a dental handpiece. Dentists reported a higher prevalence of tinnitus symptoms than expected.
Theodoroff & Folmer	Dental clinicians who regularly used high-speed handpieces had worse hearing than other groups.
Burk & Neitzel	4% of standardized 8-hr Time-Weighted Average (TWA) measurements exceeded the 85 dBA Recommended Exposure Limit. Pediatric clinics had the highest average and maximum exposures.
Kulkarni *et al*.	Sound intensities in dental settings exceeded OSHA guidelines but less than 1% of the time. Maximum recorded intensities were during non-drilling periods, attributed to suctioning.
Dierickx *et al*.	NIHL did not occur significantly more often in dentists than in controls. Dentists reported higher annoyance and more complaints related to equipment noise. All groups indicated a lack of knowledge of hearing care.
Willershausen *et al*.	Dentists' hearing slightly more impaired than controls. Statistically significant differences at 3 kHz and 4 kHz for both ears. No significant differences in bone conduction.

### Burnout

Burnout is a significant issue affecting dental professionals across various specialties and career stages. Studies have found concerning rates of burnout among dentists, dental hygienists, and dental students (Table 4). Approximately 11–16% of Spanish dentists exhibited high levels of occupational burnout, while 36.2% of Nova Scotia dental hygienists met the criteria for burnout during the COVID-19 pandemic [59]. Dental students showed even higher rates, with 40% of dental students and 38% of dental hygiene students meeting the criteria for burnout in one study [60]. The Maslach Burnout Inventory (MBI) is commonly used to assess burnout across three dimensions: emotional exhaustion (EE), depersonalization (DP), and personal accomplishment (PA) [61]. Among Nova Scotia dental hygienists, 65% scored high for EE, 34% for DP, and 24% for reduced PA [59]. A study of Turkish dental technicians found moderate levels across all three dimensions [62]. Several key factors have been identified as contributing to burnout among dental professionals. Work-related stressors play a significant role, with time pressure and productivity demands being major sources of stress. One study identified 'productivity stress' as a key dimension driving burnout among general dental practitioners, particularly those working primarily in hospitals [27]. Long working hours were also associated with increased burnout among Turkish dental technicians [62]. Work content, including repetitive tasks and a lack of variety, can contribute to burnout, with patient interactions being a significant source of stress, particularly for less experienced dentists [27]. Years of experience appear to have an inverse relationship with burnout in some studies. For example, among Iranian endodontists, those with less work experience (5 –10 years) had higher burnout scores compared to those with 10–25 years of experience [61]. Age may also play a role, with younger professionals generally reporting higher levels of burnout. Gender does not appear to be a consistent predictor of burnout, with most studies finding no significant differences between men and women [60]. The COVID-19 pandemic has had a significant impact on burnout levels among dental professionals. A study found that 36.2% met the criteria for burnout during the pandemic [59]. Dental technicians in Turkey also reported moderate levels of burnout and stress during this period [62]. The pandemic introduced new stressors, such as concerns about infection risk and adapting to new protocols [59]. Burnout is often associated with other mental health concerns. A study of dental and dental hygiene students found that 9% scored above the cut-off for moderate depressive symptoms, reporting that 6% of dental students and 9% of dental hygiene students reported clinically significant suicidal ideation, being significantly related to the lack of personal accomplishment subscale of burnout.

**Table 4:** Burnout and stress data in the included studies

Study	Main findings
Gómez-Polo *et al*.	9.8% of dentists experienced high levels of burnout Women (64.4%) showed higher EE than men (56.7%) Rural settings (70.1%) showed higher EE than urban (59.9%) Non-owners (65.6%) showed higher EE than owners (58.3%) Working alone associated with a higher risk of low PA
Haslam *et al*.	36.2% of dental hygienists met criteria for burnout during COVID-19 Contributors to burnout: time, providing dental hygiene care, expectations of dentists, physical and mental health, lack of autonomy, and COVID-19 pandemic Coping mechanisms: work-life balance, social support networks, positive work environment, physical activity EE scores were twice as high as pre-COVID-19 studies
Dikicier *et al*.	Moderate burnout levels reported (MBI-total: 37.2 ± 11.71) Moderate perceived stress levels (PSS-10 total: 21.25 ± 5.5) Long working hours increase burnout Positive correlation between perceived stress and burnout Dental technicians influenced by emotional stress due to pandemic outcomes
Hosseini *et al*.	2.9% had severe EE 4.2% exhibited intense feelings of decreased accomplishment 67.2% exhibited moderate occupational burnout 78.9% were satisfied with their job Years since graduation had an inverse correlation with burnout Recent graduates exhibited higher burnout scores
Toon *et al*.	GDPs report significantly higher stress than all other types of dentists Direct accountability for productivity Limited functional support in small business environment Combination of clinical autonomy, accountability, and relative isolation
Deeb *et al*.	Study population: Third and fourth-year dental students and first and second-year hygiene students Assessment tools: PHQ-9 and abbreviated MBI 9% of dental and dental hygiene students scored above the cut-off for moderate depressive symptoms 6% of dental students and 9% of dental hygiene students reported clinically significant suicidal ideation Depression was significantly associated with all three subscales of burnout Suicidal ideation was significantly related to the lack of personal accomplishment subscale of burnout

EE, Emotional Exhaustion; PA, Personal Accomplishment; MBI, Maslach Burnout Inventory; PSS-10, Perceived Stress Scale-10; GDP, General Dental Practitioner; PHQ-9, Patient Health Questionnaire-9; COVID-19, Coronavirus Disease 2019.

## DISCUSSION

Several reviews and systematic reviews have explored occupational health issues in dentistry, although most have focused on specific conditions [10,28,40,63,64]. Musculoskeletal disorders are particularly prevalent, with 56.4% of dentists reporting lower back pain and 58.5% reporting neck pain annually. Key risk factors include awkward postures, repetitive movements, and prolonged static positioning [10]. Occupational contact dermatitis incidence ranges from 0.6 to 6.7 per 10,000 person-years based on occupational disease registries but may be as high as 45 per 10,000 person-years in prospective studies with dental students and apprentices being particularly vulnerable, with incidence rates over 100 times higher than experienced professionals [40]. One study found that 96% of dentists surveyed did not use hearing protection devices [24]. Many dental professionals reported a lack of knowledge about hearing loss prevention. Another study found that dentists, regardless of their years of experience, reported limited knowledge about hearing care, with average scores of 1.9–2.1 out of 5 on a self-assessment knowledge scale [22]. Burnout is also common, with an overall prevalence of 13% among dentists [63]. Emotional exhaustion appears to be the most significant component, affecting 25–28% of dentists at high levels. This can lead to anxiety, depression, and even suicidal ideation in some cases [28]. Hearing loss and exposure to dental dust particles are additional occupational hazards that have been documented [64]. These reviews underscore the need for enhanced prevention strategies, particularly during the early stages of dental careers. Improving the ergonomic design of the dental workplace is crucial for reducing awkward working postures during both clinical practice and administrative tasks and alleviating MSDs [3,5,34]. Regular physical activity before and after work, back exercises, dynamic sitting, and the use of magnification loupes can significantly contribute to reducing musculoskeletal diseases and pain [6,10,33]. Health examinations should be conducted to facilitate early diagnosis and effective intervention. The use of personal protective equipment (PPE) and particle removal devices can effectively reduce adverse health effects of dust exposure [16,41]. Preventive measures are essential in managing occupational skin and pulmonary disorders in dentistry. Preventive measures are essential in managing occupational skin and pulmonary disorders in dentistry. These include environmental and personal strategies, such as improving ventilation in dental laboratories to reduce exposure to volatile compounds like methyl methacrylate (MMA), which is particularly important for protecting respiratory health[19], Proper selection and use of protective gloves, taking into account factors such as wearing time and glove material, are also crucial. Additionally, reducing the frequency of handwashing, using appropriate hand care products, and recognizing early symptoms of dermatitis or respiratory conditions are important steps. Prompt consultation with a dermatologist or pulmonologist can help ensure timely management. Where feasible, the use of high-efficiency particulate air (HEPA) filters and ultraviolet (UV) chambers in the ventilation system is recommended to enhance air quality and reduce airborne contaminants [65]. The World Health Organization recommends avoiding excessive hand washing, using soaps formulated for sensitive skin, and using alcohol-based disinfectants containing moisturizers [46]. Future research should focus on developing less harmful yet effective materials and disinfectants, as well as improving protective strategies to mitigate the impact of these essential yet potentially hazardous substances on the health of dental professionals. Regular hearing screenings and the implementation of noise reduction strategies in dental clinics could help mitigate the risk of hearing loss among dental professionals [22]. Digital noise, excluding headphones that eliminate environmental noise while allowing the passage of human voices, should be considered for the dental environment [25,64]. Future research, particularly longitudinal studies, is needed to understand better the long-term impacts of dental practice on hearing health and to develop more effective preventive strategies. Despite the potential risks, awareness and preventive measures among dental professionals appear to be limited. Studies consistently report low rates of hearing protection device usage among dentists [22,23,26]. The high prevalence of burnout among dental professionals has significant implications for both individual well-being and patient care. Recommendations emerging from the research include implementing stress management training, improving work environments, providing targeted support for early-career professionals, offering mental health screening and support, and considering policy-level interventions [27,60,66]. Teaching skills in stress management, self-care, and psychological well-being may benefit dental students and professionals [60]. Addressing factors such as time pressure, regulatory burdens, and work content can help reduce burnout [27]. Comprehensive mental health support is necessary, given the associations between burnout, depression, and suicidal ideation [59-61]. Further research is also needed to develop and evaluate interventions for reducing burnout in dental professionals, as well as to investigate the impact of provider burnout on patient care outcomes. Future research should focus on longitudinal studies to understand the progression of burnout over time and intervention studies to develop and evaluate specific strategies for prevention and mitigation. Additionally, it would be interesting to investigate the effects of recently introduced technologies, such as smartphone applications [67] and artificial intelligence [68], to understand their role in the daily work of dental professionals and their potential impact on occupational diseases. Interdisciplinary approaches, incorporating perspectives from psychology, organizational science, and public health, could enhance our understanding of this complex issue. Suitable interventions are needed to prevent occupational health issues among dental professionals, starting during the first year of their traineeship [40]. More awareness campaigns are generally needed to determine the proper measures to limit the spread of occupational diseases in dentistry. The limitations of this study are several. This scoping review aimed to provide valuable insights for practitioners and to assess the breadth and coverage of the existing literature on the topic. However, several limitations must be acknowledged. These include the significant heterogeneity of the included studies, which affects the comparability of findings; the absence of a clearly defined research question, which limits the specificity of the review’s focus; the failure to assess the risk of bias in the included articles; which raises concerns regarding the reliability of the findings; and the questionable quality of several studies, which undermines the strength of the overall evidence.

## CONCLUSION

In conclusion, occupational diseases are highly prevalent among dental professionals, significantly impacting their health and career longevity. Musculoskeletal disorders, particularly affecting the lower back and neck, are extremely common. Occupational contact dermatitis is also prevalent, with dental students and apprentices being particularly vulnerable. Dental professionals face risks of hearing loss and exposure to harmful dust particles. Burnout syndrome is a considerable issue, with emotional exhaustion being the most significant component. A majority of dentists report moderate to severe work-related stress, which can lead to anxiety, depression, and, in some cases, suicidal ideation. These high prevalence rates underscore the urgent need for improved prevention strategies, particularly in the early stages of dental careers.

## Data Availability

Further data is available from the corresponding author upon reasonable request.

## Personal thanks

The publication of this paper was supported by Carol Davila University of Medicine and Pharmacy through the Doctoral School.

## References

[1] Occhionero V, Korpinen L, Gobba F (2014). Upper limb musculoskeletal disorders in healthcare personnel. Ergonomics.

[2] Haas Y, Naser A, Haenel J, Fraeulin L, Holzgreve F, Erbe C (2020). Prevalence of self-reported musculoskeletal disorders of the hand and associated conducted therapy approaches among dentists and dental assistants in Germany. PLoS One.

[3] Lietz J, Ulusoy N, Nienhaus A (2020). Prevention of musculoskeletal diseases and pain among dental professionals through ergonomic interventions: a systematic literature review. Int J Environ Res Public Health.

[4] Eyvazlou M, Asghari A, Mokarami H, Bagheri Hosseinabadi M, Derakhshan Jazari M, Gharibi V (2021). Musculoskeletal disorders and selecting an appropriate tool for ergonomic risk assessment in the dental profession. Work.

[5] Gandolfi MG, Zamparini F, Spinelli A, Risi A, Prati C (2021). Musculoskeletal disorders among Italian dentists and dental hygienists. Int J Environ Res Public Health.

[6] Kapitán M, Hodačová L, Čermáková E, Machač S, Schmidt J, Pilbauerová N (2021). The development of musculoskeletal disorders during undergraduate dentistry studies—a long-term prospective study. Int J Environ Res Public Health.

[7] Gupta A, Bhat M, Mohammed T, Bansal N, Gupta G (2014). Ergonomics in dentistry. Int J Clin Pediatr Dent.

[8] Arheiam A, Ingafou M (2015). Self-reported occupational health problems among Libyan dentists. J Contemp Dent Pract.

[9] Soo SY, Ang WS, Chong CH, Tew IM, Yahya NA (2023). Occupational ergonomics and related musculoskeletal disorders among dentists: a systematic review. Work.

[10] Lietz J, Kozak A, Nienhaus A (2018). Prevalence and occupational risk factors of musculoskeletal diseases and pain among dental professionals in Western countries: a systematic literature review and meta-analysis. PLoS One.

[11] Mulimani P, Hoe VC, Hayes MJ, Idiculla JJ, Abas AB, Karanth L (2018). Ergonomic interventions for preventing musculoskeletal disorders in dental care practitioners. Cochrane Database Syst Rev.

[12] Plessas A, Bernardes Delgado M (2018). The role of ergonomic saddle seats and magnification loupes in the prevention of musculoskeletal disorders. A systematic review. Int J Dent Hyg.

[13] Japundžić I, Lugović-Mihić L (2019). Skin reactions to latex in dental professionals—first Croatian data. Int J Occup Saf Ergon.

[14] Bishop S, Roberts H (2020). Methacrylate perspective in current dental practice. J Esthet Restor Dent.

[15] Vaiude AP, Jawdekar A, Mistry LN (2024). Prevalence of latex allergy in dental professionals: a systematic review and meta-analysis. Community Dent Health.

[16] Ergün D, Ergün R, Ozdemir C, Oziş TN, Yilmaz H, Akkurt I (2014). Pneumoconiosis and respiratory problems in dental laboratory technicians: analysis of 893 dental technicians. Int J Occup Med Environ Health.

[17] Alıcı N, Çımrın A, Coşkun Beyan A (2016). Pneumoconiosis in different sectors and their differences in Turkey. Tuberk Toraks.

[18] Di Lorenzo L, Inchingolo F, Pipoli A, Cassano F, Maggiore ME, Inchingolo AM (2022). Mixed-dust pneumoconiosis in a dental technician: a multidisciplinary diagnosis case report. BMC Pulm Med.

[19] Okamoto M, Tominaga M, Shimizu S, Yano C, Masuda K, Nakamura M (2017). Dental Technicians’ Pneumoconiosis. Intern Med.

[20] Burk A, Neitzel RL (2016). An exploratory study of noise exposures in educational and private dental clinics. J Occup Environ Hyg.

[21] Al-Omoush SA, Abdul-Baqi KJ, Zuriekat M, Alsoleihat F, Elmanaseer WR, Jamani KD (2020). Assessment of occupational noise-related hearing impairment among dental health personnel. J Occup Health.

[22] Dierickx M, Verschraegen S, Wierinck E, Willems G, van Wieringen A (2021). Noise disturbance and potential hearing loss due to exposure of dental equipment in Flemish dentists. Int J Environ Res Public Health.

[23] Khaimook W, Suksamae P, Choosong T, Chayarpham S, Tantisarasart R (2014). The prevalence of noise-induced occupational hearing loss in dentistry personnel. Workplace Health Saf.

[24] Myers J, John AB, Kimball S, Fruits T (2016). Prevalence of tinnitus and noise-induced hearing loss in dentists. Noise Health.

[25] Willershausen B, Callaway A, Wolf TG, Ehlers V, Scholz L, Wolf D (2014). Hearing assessment in dental practitioners and other academic professionals from an urban setting. Head Face Med.

[26] Theodoroff SM, Folmer RL (2015). Hearing loss associated with long-term exposure to high-speed dental handpieces. Gen Dent.

[27] Toon M, Collin V, Whitehead P, Reynolds L (2019). An analysis of stress and burnout in UK general dental practitioners: subdimensions and causes. Br Dent J.

[28] Pontes CC, Stanley K, Molayem S (2024). Understanding the dental profession's stress burden: prevalence and implications. Compend Contin Educ Dent.

[29] Munn Z, Peters MDJ, Stern C, Tufanaru C, McArthur A, Aromataris E (2018). Systematic review or scoping review? Guidance for authors when choosing between a systematic or scoping review approach. BMC Med Res Methodol.

[30] Tricco AC, Lillie E, Zarin W, O’Brien K, Colquhoun H, Kastner M (2016). A scoping review on the conduct and reporting of scoping reviews. BMC Med Res Methodol.

[31] Khalil H, Peters M, Tricco A, Pollock D, Alexander L, McInerney P (2020). Conducting high quality scoping reviews: challenges and solutions. J Clin Epidemiol.

[32] Ohlendorf D, Naser A, Haas Y, Haenel J, Fraeulin L, Holzgreve F (2020). Prevalence of musculoskeletal disorders among dentists and dental students in Germany. Int J Environ Res Public Health.

[33] Hodacova L, Sustova Z, Cermakova E, Kapitan M, Smejkalova J (2015). Self-reported risk factors related to the most frequent musculoskeletal complaints among Czech dentists. Ind Health.

[34] Šćepanović D, Klavs T, Verdenik I, Oblak Č (2019). The prevalence of musculoskeletal pain of dental workers employed in Slovenia. Workplace Health Saf.

[35] Aljanakh M (2024). Musculoskeletal disorders among dental assistants: a cross-sectional study. BMC Musculoskelet Disord.

[36] Al-Huthaifi BH, Al Moaleem MM, Alwadai GS, Abou Nassar J, Sahli AAA, Khawaji AH (2023). High prevalence of musculoskeletal disorders among dental professionals: a study on ergonomics and workload in Yemen. Med Sci Monit.

[37] Botha PJ, Chikte U, Barrie R, Esterhuizen TM (2014). Self-reported musculoskeletal pain among dentists in South Africa: a 12-month prevalence study. SADJ.

[38] Maghsoudipour M, Hosseini F, Coh P, Garib S (2021). Evaluation of occupational and non-occupational risk factors associated with carpal tunnel syndrome in dentists. Work.

[39] Alhusain FA, Almohrij M, Althukeir F, Alshater A, Alghamdi B, Masuadi E (2019). Prevalence of carpal tunnel syndrome symptoms among dentists working in Riyadh. Ann Saudi Med.

[40] Larese Filon F, Pesce M, Paulo MS, Loney T, Modenese A, John SM (2021). Incidence of occupational contact dermatitis in healthcare workers: a systematic review. J Eur Acad Dermatol Venereol.

[41] Kucharczyk M, Słowik-Rylska M, Cyran-Stemplewska S, Gieroń M, Nowak-Starz G, Kręcisz B (2021). Acrylates as a significant cause of allergic contact dermatitis: new sources of exposure. Postepy Dermatol Alergol.

[42] Bernhoft RA (2012). Mercury toxicity and treatment: a review of the literature. J Environ Public Health.

[43] Bodnar DC, Burlibașa L, Varlan C, Marcov N, Georgescu SR, Marcov CE (2009). Mercury, biocompatibility and its impact on environment. J Med Invest.

[44] Berlin M (2020). Mercury in dental amalgam: a risk analysis. Neurotoxicology.

[45] Al-Asmar AA, Ha Sabrah A, Abd-Raheam IM, Ismail NH, Oweis YG (2023). Clinical evaluation of reasons for replacement of amalgam vs composite posterior restorations. Saudi Dent J.

[46] Ramos L, Cabral R, Gonçalo M (2014). Allergic contact dermatitis caused by acrylates and methacrylates—a 7-year study. Contact Dermatitis.

[47] Stoeva IL (2020). Work-related skin symptoms among Bulgarian dentists. Contact Dermatitis.

[48] Heratizadeh A, Werfel T, Schubert S, Geier J (2018). Contact sensitization in dental technicians with occupational contact dermatitis: data of the Information Network of Departments of Dermatology (IVDK) 2001–2015. Contact Dermatitis.

[49] Soykut B, Erdem O, Yalçın C, Üstündağ A, Duydu Y, Akay C (2020). Occupational exposure of dental technicians to methyl methacrylate: genotoxicity assessment. Mutat Res Genet Toxicol Environ Mutagen.

[50] Ergün R, Ergün D, Evcik E, Ergan B (2017). Evaluation of dental technician's pneumoconiosis using chest X-ray and HRCT: correlation between radiological and functional findings. Turk J Med Sci.

[51] Kahraman H, Koksal N, Cinkara M, Ozkan F, Sucakli MH, Ekerbicer H (2014). Pneumoconiosis in dental technicians: HRCT and pulmonary function findings. Occup Med (Lond).

[52] Ergün D, Ergün R, Evcik E, Nadir Öziş T, Akkurt İ (2016). The relation between the extent of radiological findings and respiratory functions in pneumoconiosis cases of dental technicians who are working in Ankara. Tuberk Toraks.

[53] Stoeva I (2021). Respiratory symptoms of exposure to substances in the workplace among Bulgarian dentists. Community Dent Oral Epidemiol.

[54] Gonçalves CG, Santos L, Lobato D, Ribas A, Lacerda AB, Marques J (2015). Characterization of hearing thresholds from 500 to 16,000 Hz in dentists: a comparative study. Int Arch Otorhinolaryngol.

[55] Al-Rawi NH, Al Nuaimi AS, Sadiqi A, Azaiah E, Ezzeddine D, Ghunaim Q (2019). Occupational noise-induced hearing loss among dental professionals. Quintessence Int.

[56] Gavrila L, Mircea LJZ (2001). Chromatin and chromosomal fine structure in spermatogenesis of some species of amphibians. J Morphol Sci.

[57] Rabinowitz PM (2000). Noise-induced hearing loss. Am Fam Physician.

[58] Kulkarni E, Abdallah Y, Hanseman D, Krishnan DG (2018). How much noise is an oral and maxillofacial surgeon exposed to?. J Oral Maxillofac Surg.

[59] Haslam SK, Wade A, Macdonald LK, Johnson J, Rock LD (2022). Burnout syndrome in Nova Scotia dental hygienists during the COVID-19 pandemic: Maslach Burnout Inventory. Can J Dent Hyg.

[60] Deeb GR, Braun S, Carrico C, Kinser P, Laskin D, Golob Deeb J (2018). Burnout, depression and suicidal ideation in dental and dental hygiene students. Eur J Dent Educ.

[61] Hosseini B, Manochehrifar H, Shahravan A, Yazdani A, Malek Mohammadi T, Mohammadzadeh I (2024). Evaluation of occupational burnout and job satisfaction among endodontists in Iran. Iran Endod J.

[62] Dikicier S, Korkmaz C, Atay A, Yilmaz MD (2023). Evaluation of burnout and stress perception levels of Turkish dental laboratory technicians during the COVID-19 pandemic. Ann Med.

[63] Moro JDS, Soares JP, Massignan C, Oliveira LB, Ribeiro DM, Cardoso M (2022). Burnout syndrome among dentists: a systematic review and meta-analysis. J Evid Based Dent Pract.

[64] Hartland JC, Tejada G, Riedel EJ, Chen AH, Mascarenhas O, Kroon J (2023). Systematic review of hearing loss in dental professionals. Occup Med (Lond).

[65] Ding J, Li J, Qi J, Fu L (2023). Characterization of dental dust particles and their pathogenicity to respiratory system: a narrative review. Clin Oral Investig.

[66] Long H, Li Q, Zhong X, Yang L, Liu Y, Pu J (2023). The prevalence of professional burnout among dentists: a systematic review and meta-analysis. Psychol Health Med.

[67] Pascadopoli M, Zampetti P, Nardi MG, Pellegrini M, Scribante A (2023). Smartphone Applications in Dentistry: A Scoping Review. Dent J (Basel).

[68] Lee J, Bae SR, Noh HK (2023). Commercial artificial intelligence lateral cephalometric analysis: part 1—the possibility of replacing manual landmarking with artificial intelligence service. J Clin Pediatr Dent.

